# Chicago Medical Society, June Meeting

**Published:** 1884-09

**Authors:** 


CHICAGO MEDICAL SOCIETY.

Regular meeting, 16th June, 1884. The President, Dr. D. A. K. Steele, in the chair.

The following papers, essays and reports of cases were presented: The Significance of Jaundice in Diagnosis, by Dr. Wm. E. Quine. The Effect of Noises upon Certain Forms of Deafness, Dr. G. F. Hawley. A Case of Genito-Urinary Surgery and Median Lithotomy, Dr. William L. Axford. Pathological Specimens of Papillomatous Ovarian Cystic Tumors, Dr. Charles T. Parkes.

The following is a brief synopsis of Dr. Quine's paper on "The Significance of Jaundice in Diagnosis:"

1. Jaundice occurring suddenly, in apparent health, and painlessly, is usually of emotional origin, and transitory.

2. When it depends on disease or injury of the brain, acute atrophy of the liver, snake poison, or an infectious fever, it is always associated with mental disturbance.

3. If it be attended with fever, and well marked, it is secondary to inflammation of biliary passages, pneumonia, toxæmia, or infective inflammation of the portal vein.

4. If it occur suddenly, and is preceded by paroxysmal pain and vomiting, it is caused, nine times out of ten, by biliary calculi.

5. If it be preceded by typical symptoms of gastro-duodenal inflammation, it is obviously of catarrhal origin.

6. Impassable obstruction of the common duct is shown by great intensity of jaundice, clay-colored stools, and in recent cases by distension of the gall-bladder.

7. Jaundice, caused by sudden obstruction of the biliary passages, is always associated with paroxysmal pain and nausea, but there is no means of ascertaining the nature of the obstructing body, except its discovery in the stools.

8. In the rare cases of sudden obstruction by cancerous, hydatid and aneurismal tumors, there is almost always a history of impaired health, enlargement and deformity of the liver, ascites, etc., which, aided by the revelations of physical exploration, will lead to correct differentiation.

9. Sudden return of normal coloring to fæces confirms the diagnosis of obstruction.

10. Occlusion of the cystic duct may be attended with as much pain, nausea, and distension of the gall-bladder, as occlusion of the common duct, but there is no jaundice. In occlusion of the hepatic duct, the same symptoms are present, including jaundice, and excluding distension of the gall-bladder It is often impossible to distinguish between occlusion of the hepatic and the common duct. The former is rare because the duct increases in size from above downward.

11. If jaundice persist after the symptoms of biliary colic or catarrhal inflammation have a month since disappeared; or if jaundice have disappeared after a biliary colic, to return slowly and painlessly, it may be assumed that stricture of the duct has resulted from inflammatory thickening, adhesion of walls, or cicatrization of an ulcer.

12. A history of repeated attacks points to the probability of gall stones.

13. If jaundice come on slowly, without antecedent colic, or catarrh, and without attendant evidence of impaired health or portal obstruction, it is probably caused either by pressure upon the duct, or by the growth of a tumor within its walls. The pressing body, when large enough, may be readily appreciated, as in the case of pregnancy, ovarian tumor, aneurism, distended colon, etc., but when it is small, or constituted by enlargement of lymphatics in the fissure of the liver, it is apt to escape detection.

14. Slight but persistent jaundice may be due to incomplete occlusion of the common duct, or to complete occlusion of a branch of the hepatic; but usually it is found associated witheither valvular disease of the heart, some disease of the lungs which obstructs circulation, or cirrhosis of the liver.

15. If ascites be associated with it, the disease is either cirrhosis or cancer of the liver. If the liver be abnormally small, the disease is cirrhosis; if it be large, the disease is either hypertrophic cirrhosis or cancer. Differentiation between the two is seldom attended with difficulty.

16. Absence of jaundice does not imply absence of hepatic disease, since the liver may be destroyed by disease, or extirpated by operation, without jaundice ensuing.

17. It is not a prominent symptom of hepatitis, if catarrhal inflammation of biliary passages be excluded. It is not characteristic of hepatic abscess, where, at most, mere muddiness of the complexion is usually seen. These affections are rare in temperate climates, and when encountered, are generally found to be secondary to direct injury of the liver, or to infective inflammation of the portal vein. It is not a symptom of waxy or fatty liver, or of hydatids, excepting as an extraordinary complication.

The paper was ably discussed by Drs. G. C. Paoli, S. H. Stevenson, J. J. M. Angear, A. R. Jackson, J. H. Etheridge,

C. T. Fenn, and R. Tilley. The discussion was closed by Dr. Wm. E. Quine.

A paper upon "The Effect of Noises upon Certain Forms of Deafness" was read by Dr. G. F. Hawley. This paper has already appeared in a former number of the JOURNAL.

Comments upon the paper were made by Professor S. J. Jones and Dr. R. Tilley, and the discussion closed by Dr. Hawley.

